# Comparison of Sensory Qualities in Eggs from Three Breeds Based on Electronic Sensory Evaluations

**DOI:** 10.3390/foods10091984

**Published:** 2021-08-25

**Authors:** Xiaoguang Dong, Libing Gao, Haijun Zhang, Jing Wang, Kai Qiu, Guanghai Qi, Shugeng Wu

**Affiliations:** Risk Assessment Laboratory of Feed Derived Factors to Animal Product Quality Safety (Beijing) of Ministry of Agriculture & Rural Affairs, Institute of Feed Research, Chinese Academy of Agricultural Sciences, Beijing 100081, China; 82101195208@caas.cn (X.D.); 82101195206@caas.cn (L.G.); zhanghaijun@caas.cn (H.Z.); wangjing@caas.cn (J.W.); qiukai@caas.cn (K.Q.); wushugeng@caas.cn (S.W.)

**Keywords:** egg, sensory, electronic nose, electronic tongue, discrimination

## Abstract

The present study was conducted on three commercial laying breeder strains to evaluate differences of sensory qualities, including texture, smell, and taste parameters. A total of 140 eggs for each breed were acquired from Beinong No.2 (B) laying hens, Hy-Line Brown (H) laying hens, and Wuhei (W) laying hens. Sensory qualities of egg yolks and albumen from three breeds were detected and discriminated based on different algorithms. Texture profile analysis (TPA) showed that the eggs from three breeds had no differences in hardness, adhesiveness, springiness, and chewiness other than cohesiveness. The smell profiles measured by electronic nose illustrated that differences existed in all 10 sensors for albumen and 8 sensors for yolks. The taste profiles measured by electronic tongue found that the main difference of egg yolks and albumen existed in bitterness and astringency. Principal component analysis (PCA) successfully showed grouping of three breeds based on electronic nose data and failed in grouping based on electronic tongue data. Based on electronic nose data, linear discriminant analysis (LDA), fine k-nearest neighbor (KNN) and linear support vector machine (SVM) were performed to discriminate yolks, albumen, and the whole eggs with 100% classification accuracy. While based on electronic tongue data, the best classification accuracy was 96.7% for yolks by LDA and fine tree, 88.9% for albumen by LDA, and 87.5% for the whole eggs by fine KNN. The experiment results showed that three breeds’ eggs had main differences in smells and could be successfully discriminated by LDA, fine KNN, and linear SVM algorithms based on electronic nose.

## 1. Introduction

Eggs are an inexpensive supplier of high quality protein, balanced vitamins, and minerals relative to their low calorie content [1]. Sensory qualities, influencing egg overall quality and marketing, were studied in textures and flavors as representative indexes. Flavors as important sensory qualities are characterized by smells and tastes [2]. Egg sensory qualities are influenced by external conditions of feeding, mainly diets supplementation of seaweeds [3,4], linseed [5,6], fish oil [7,8], hempseed and hempseed oil [9], canola meal and flaxseed oil [10], arachidonic acid [11], and conjugated linoleic acid [12]. Rearing systems [13] and storage conditions [14] also could affect egg sensory qualities. Egg breed, as an internal factor to determine egg composition and content, influences egg sensory qualities. Egg sensory qualities of five different brown egg breeds and one white egg breed were tested by artificial sensory evaluation [15]. Volatile compounds of white Leghorn, Hy-Line Brown (H), and Jing Fen hatching eggs were compared by a gas chromatography-mass spectroscopy (GC-MS) [16]. H laying hens as a common breed in production are widely used as an experimental material in egg researches [17]. Beinong No.2 (B) laying hens as a local breed are feed-saving, small hybrid laying hens. Wuhei (W) laying hens, known as “five black and one green,” have the characteristics of black hair, black skin, black meat, black bone, black viscera, and green eggshell. As mentioned above, B, H, and W laying hens as three different breeds would have differences in sensory qualities. The major constituents of egg yolk are proteins and lipids, while the major constituents of albumen are water and protein [18]. The yolks and albumen from different breeds would have different degree diversity characteristics in sensory qualities. The sensory qualities include texture, smell, and taste parameters. Compared with artificial sensory evaluation [19,20], instrumental sensory measurements could provide more objective and rapid evaluation. Texture profile analysis (TPA) by a texture analyzer simulates the way food feels in the mouth, including hardness, adhesiveness, cohesiveness, springiness, and chewiness [21]. Texture properties were measured to compare nutraceutical egg products with omega-3-rich oils [22]. For smell analysis, a GC-MS was used to separate and identify volatile compounds, although there are disadvantages, including expense, complexity, and time-consuming nature [23]. An electronic nose equipped with artificial sensors could detect volatile compounds with higher sensitivity and accuracy compared to the human nose. The electronic nose provides different responses to different smells in food samples and has fast and non-destructive advantages in detecting the integral smells instead of individual smells by GC-MS. In egg analysis, the electronic nose was used to detect fertilization status [24], TVB-N content [25], storage time [26], and egg freshness [27]. Regarding the taste components, an electronic tongue system could characterize the slight differences in tastes without subjective factors and judgments and prove a powerful tool to distinguish taste profiles [28]. The electronic tongue, based on the organizational principles of biological sensory systems, was mainly applied in taste quantification and recognition [29]. The taste sensor was applied to the discrimination of different kinds of food [30]. Electronic nose and electronic tongue were used to analyze sensory differences of livestock products [31], aquatic products [32,33], and milk [34]. There was little research available on egg smells and tastes based on both electronic nose and electronic tongue. 

Electronic nose and electronic tongue instrumental systems were designed to crudely mimic human smell and taste sensory organs [35]. Complex data sets from electronic nose and electronic tongue signals were usually combined with multivariate statistics for classification, discrimination, recognition, and identification of samples [36]. Principal component analysis (PCA) as a multi-variate data analysis method reduces the dimensionality of the data and makes the analysis simpler with smaller set of variables [37]. Electronic tongue combined with PCA was use to separate tea [38], vegetable oils [39], and stored rice [40]. Electronic nose data processed by PCA successfully grouped eggs from seven different species, including duck, free-range chicken, silky chicken, quail, pigeon, goose, and chicken [41]. Though geometrical distribution of the PCA plot, it could not be directly used as a tool for discrimination. Therefore, classification algorithms, including k-nearest neighbor (KNN), linear discriminant analysis (LDA) [42], and support vector machine (SVM) and decision tree classification [43], were performed in pattern recognition and spatial data processing. According to the distance between them and the selected point, the LDA classes data points are projected onto a straight line to reduce the dimension of the data set. The KNN algorithm checks the distance between a test sample and a training sample. The SVM, powerful in handling the problem with small, non-linear, and high-dimensional data sets, draws a line between different data point clusters and groups them into some classes. The decision tree is computed by decomposing the data set into smaller and smaller subsets according to different criteria. PCA was reported to get better results than LDA to distinguish pure and adulterated honey samples based on electronic nose [44]. Based on electronic nose data, LDA could classify eggs based on storage time, and SVM was applied to build prediction models of yolk index with square correlation coefficient of 0.9641 in a training set and 0.8339 in a testing set [45]. 

The aim of this study was to compare textures, smells, and tastes of eggs from three breeds. To get a more detailed look at internal sensory qualities of eggs, yolks and albumen of three breeds were analyzed and classified separately based on electronic sensory evaluations combined with classification algorithms. The results could provide theoretical basis and technical support to discriminate different breeds of eggs. 

## 2. Material and Methods

### 2.1. Samples Preparation

A total of 140 eggs for each breed were acquired from B, H, and W laying hens (30-week-old) under corn-based feed with all nutrients in Zhuozhou experiment base of Chinese Academy of Agricultural Sciences. Eggs were available in the laboratory 24 h after being laid and stored at 20 ± 1 °C. Ten fresh, uncooked eggs were used for egg quality analysis. Ten cooked eggs were used for TPA. The electronic nose was adopted to test 48 eggs with three measurement replicates of six pooled samples with eight birds each. Nine pooled yolk and albumen samples, each consisting of eight cooked eggs, were analyzed for electronic tongue analysis. Yolks and albumen of the cooked eggs, boiled in the Egg Cooker (Model ZDQ-B07C3, Bear Electric Co., Ltd., Foshan, China) for 12 min, were used for TPA, electronic nose, and electronic tongue analysis. 

### 2.2. Egg Quality Analysis

Egg quality, including egg weight, albumen height, yolk color, and Haugh unit indexes, were detected by a multifunctional egg multi tester (Model EA-01, ORKA Food Technology Ltd., Ramat Hasharon, Israel). 

### 2.3. Texture Profile Analysis (TPA)

The cooked yolks and cylindrical cooked albumen from a cylindrical sampler with 2-cm diameter were measured using a texture analyzer (Model TMS-PRO, Food Technology Corporation, Sterling, VA, USA). TPA was performed with trigger force of 0.05 N, two-cycle deformation of 40%, and a speed of 30 mm/min. From the resulting force-time curves, hardness, adhesiveness, cohesiveness, springiness, and chewiness were determined [46]. 

### 2.4. Electronic Nose Analysis

Yolk and albumen smells were analyzed by an electronic nose (Model PEN3, Airsense Company, Schwerin, Germany) equipped with 10 metal oxide semiconductor sensors. As shown in Table 1, 10 sensors monitoring smells in the mixture based on the time response curve are sensitive to different volatile compounds [25]. A total of 20 g of cooked yolks or albumen were put in an 15-mL sealed vial, and the parameters of the electronic nose were as follows: sample interval, 1 s; flush time, 200 s; zero point trim time, 10 s; pre-sampling time, 5 s; measurement time, 120 s; chamber flow, 300 mL/min; initial injection flow, 300 mL/min [41,45]. 

### 2.5. Electronic Tongue Analysis

An electronic tongue system (Model SA402B, Insent Intelligent Sensor Technology, Inc., Atsugi-Shi, Japan) equipped with an array of potentiometric chemical sensors (AE1, C00, AAE, CT0, and CA0) was employed to characterize yolk and albumen tastes. A total of 40 g of cooked yolks or albumen were soaked in 160 mL of water, mixed for 10 min of ultrasonic, and centrifugated at 5000 r/min speed for 10 min before filtering. The filtrate was used for electronic tongue determination. For the test of electronic tongue, the sampling time of each sample was 120 s, and the cleaning time was 120 s.

### 2.6. Statistical Analysis

Experimental data were subjected to one-way analysis of variance (ANOVA) using SPSS 17.0. A significant difference was used at 0.05 probability level (*p* < 0.05). PCA was performed by Origin 2019b. The LDA, fine KNN, and linear SVM and fine tree were used to classify the yolks, albumen, and whole egg of three breeds. All computations were performed by Classification Learner in MATLAB App Designer of MATLAB software (2018a, Mathworks Inc., Natick, MA, USA) under the Windows 10 system. K-fold cross validation was used to divide the dataset into training and testing set. Predicted class in models was calculated and compared with true class with 1 for W eggs, 2 for B eggs, and 3 for H eggs. Classification abilities of different algorithms were shown by classification accuracy and confusion matrix.

## 3. Results and Discussion 

### 3.1. Egg Quality Analysis

The egg quality of B, H, and W eggs is shown in Table 2. The yolk color value of H eggs with 6.50 was significantly lower than those of B eggs with 9.80 and W eggs with 9.10 (*p* < 0.05). The W eggs had a lower weight with 44.97 g than B with 55.45 g and H eggs with 54.86 g (*p* < 0.05). The egg weight of Lohmann Brown laying hens was about 59.44 g [47]. The weight of Issa Brown pullet eggs was reported ranging from 65.77 to 62.62 g [48]. H eggs fed by *Camelina sativa* oil at week 35 had weights from 61.00 to 61.30 g [49]. Egg weights are reported to vary with breeds, ages, and diets. The albumen height of H eggs was 6.38 mm, which was significantly higher than that of W eggs (*p* < 0.05). Eggs from different breeds vary in yolk color, egg weight, and albumen height. While HU was more than 72 for all breeds, representing egg freshness, it showed no significant differences in B, H, and W eggs (*p* > 0.05), indicating that egg freshness of the three breeds of eggs was consistent. The HU has four grades in the international standard from the U.S. Department of Agriculture. The AA grade with the HU value greater than 72 represents that the egg is fresh and suitable for consumers. Grade A with HU from 60 to 72 indicates that the egg is edible. Grade B with HU from 30 to 60 means the egg is no longer fit to eat. Grade C with HU less than 30 indicates the egg is inedible. For fresh and edible eggs, the HU is usually more than 72. Egg quality did not reveal any significant differences in HU, in agreement with previous researches [47]. The consistent egg freshness made sure that following researches about sensory qualities were mainly influenced by breeds.

### 3.2. Texture Profile Analysis (TPA)

For egg yolk textures in Table 3, hardness, adhesiveness, springiness, and chewiness were not significantly different in three breeds (*p* > 0.05). However, the cohesiveness of B yolks was higher than that of W yolks (*p* < 0.05). For albumen textures, Table 4 reveals that there were no significant differences (*p* > 0.05) in hardness, adhesiveness, springiness, and chewiness from the three breeds of eggs. The W albumen had higher cohesiveness than B and H albumen (*p* < 0.05). The TPA results illustrated that hardness, representing the maximum force required to compress the sample; adhesiveness, indicating work necessary to pull probe away from the sample; springiness, meaning ability of sample to recover its original form after the deforming force is removed; and chewiness, relating to the work needed to chew a solid sample to a steady state of swallowing, had no significant differences in egg yolks and albumen from three breeds (*p* > 0.05). The cohesiveness, corresponding to the extent to which the sample can be deformed before rupture, was significantly different in egg yolks and albumen from the three breeds (*p* < 0.05) [22].

### 3.3. Electronic Nose Analysis

**Figure 1:**
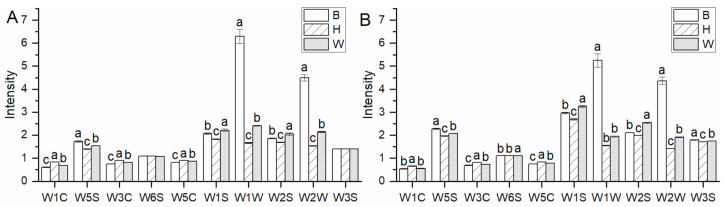
Electronic nose histograms with 10 sensors of egg yolks and albumen from three breeds. (**A**) Electronic nose histogram of yolks. (**B**) Electronic nose histogram of albumen. B, Beinong No.2; H, Hy-Line Brown; W, Wuhei. a,b,c means a sensor with no common letters significantly differs (*p* < 0.05) (*n* = 18).

Electronic nose detection, with good reproducibility and repeatability, is a simple, quick, non-destructive, and specific technique. Electronic nose is adopted for measuring the volatile fingerprint based on the time responses of sensor arrays but could not identify flavor compounds as precisely as GC-MS [50]. Electronic nose could detect the characteristic differences in the flavor profiles in yolks and albumen. As shown in Figure 1, the values of electronic nose histogram were on behalf of the average signal variation for every sensor. For egg yolks in Figure 1A, sensors W1W, W2W, W1S, W2S, and W5S displayed higher values than the rest of the sensors, with W1W of B eggs exhibiting the highest value. This indicated that these five sensors had larger proportions in egg yolk tastes. It was clear that eight sensors, all except for W6S and W3S, had significant differences in egg yolks from three breeds (*p* < 0.05). For egg albumen in Figure 1B, sensors W1W, W2W, W1S, W2S, and W5S also exhibited higher values than the rest, with W1W of B eggs getting the highest value. Egg albumen had the same taste characteristics as egg yolks. All 10 sensors had differences in egg albumen from three breeds (*p* < 0.05). According to above results, W1W, sensitive to sulfur compounds; W2W, sensitive to aromatic, sulfur organic; W1S, sensitive to methane; W2S, sensitive to alcohol, aromatic; and W5S, sensitive to nitrogen oxides, were the main flavor components of egg yolks and albumen from the three breeds. The most volatile compounds measured by electronic nose showed significant differences in egg yolks and albumen from three breeds, in which sulfur and aromatic compounds of B egg yolks and albumen shown in W1W and W2W had more obvious spikes than H and W yolks and albumen (*p* < 0.05).

**Figure 2:**
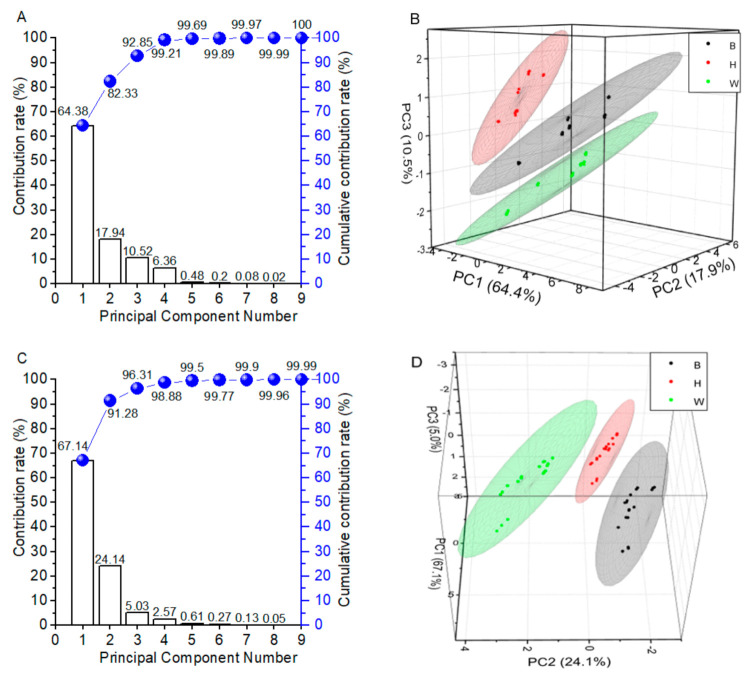
Principal component analysis (PCA) of egg yolks and albumen by electronic nose (*n* = 18). (**A**) Contribution rate and cumulative contribution rate of yolks. (**B**) PCA score plot of yolks. (**C**) Contribution rate and cumulative contribution rate of albumen. (**D**) PCA score plot of albumen. B, Beinong No.2; H, Hy-Line Brown; W, Wuhei.

PCA was used to reduce dimensionality with a minimum loss of information, transform the original variable data, and eliminate the overlapping parts in the coexistence of a great deal of information [51]. PCA could present the distinction among three breeds’ eggs and indicate the relative importance of sensors in explaining the variance of breeds. It is often found that the first few principal components account for most of the information present in the original data set. For yolks in Figure 2A, the contribution rate of the first principal component (PC1), the second principal component (PC2), and the third principal component (PC3) were 64.4%, 17.9%, and 10.5%, respectively. The total accumulative contribution rate of PC1, PC2, and PC3 reached 92.8%, which proved that PC1, PC2, and PC3 could be used as representatives for subsequent analysis. As displayed in Figure 2B, it was clear that egg yolks from three breeds were clearly separated from each other by PCA. For albumen in Figure 2C, the total variance explained by the PCA was 96.3%, where PC1, PC2, and PC3 explained 67.1%, 24.1%, and 5.0%, respectively. A good separation of B, H, and W albumen was also obtained. PCA projects the data onto fewer dimensions and hence was chosen to exploit the relationships between the variables. PCA is an unsupervised method for grouping egg yolks and albumen from the three breeds based on the inherent similarity or dissimilarity of their chemical information without prior knowledge of egg breeds. The PCA was reported to discriminate different storage time, as the freshness quality was based on electronic nose data [26].

**Figure 3:**
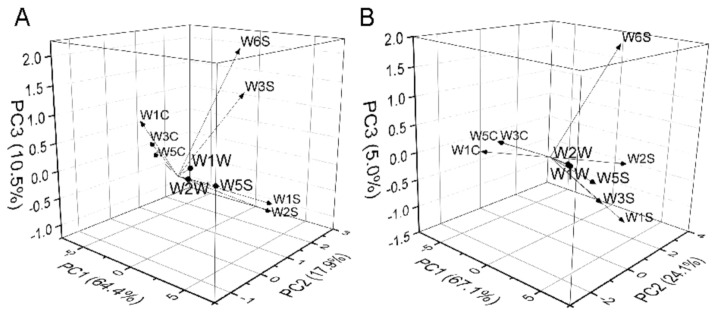
The load coefficient diagrams of electronic nose by principal component analysis (PCA). (**A**) The load coefficient diagrams for yolks. (**B**) The load coefficient diagrams for albumen.

PCA load coefficients, which revealed compounds in charge of yolk and albumen smells, are illustrated in Figure 3 and Table 5. For egg yolks, sensors W5S, W1W, and W2W had load coefficients more than 0.35 in PC1, which indicates that nitrogen oxides, aromatic, and sulfur organic had the main positive contribution. The load coefficients of sensors W1C, W3C, and W5C were less than −0.35 in PC1, which indicates that aromatic, ammonia, and alkane had the main negative contribution in yolks. In PC2, sensors W2S and W6S had higher load coefficients of 0.57 and 0.49. In PC3, sensor W6S had the highest load coefficient of 0.78. For egg albumen, only sensor W5S had a load coefficient more than 0.35 in PC1, which indicates that nitrogen oxides had the main positive contribution. Similar to egg yolks, sensors W1C, W3C, and W5C for egg albumen had load coefficients less than −0.35 in PC1, which indicates that aromatic, ammonia, and alkane also had the main negative contribution in egg albumen. In PC2 and PC3, W2S and W6S had higher load coefficients, consistent with previous yolk results. On the whole, sensors W5S, W1C, W3C, W5C, W2S, and W6S had higher influence in PCA of egg yolks and albumen from three breeds. On the other hand, sensors W5S, W1C, W3C, and W5C, W2S, and W1S had greater effect on discrimination of Brown Hy-Line eggs based on storage periods [25]. Only one different sensor interpreted that main sensors would change with egg breeds and indexes.

### 3.4. Electronic Tongue Analysis

**Figure 4:**
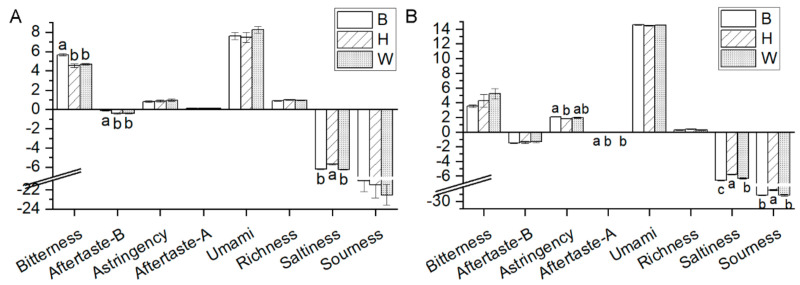
Electronic tongue histograms of egg yolks and albumen from three breeds. (**A**) Electronic tongue histogram of yolks. (**B**) Electronic tongue histogram of albumen. B, Beinong No.2; H, Hy-Line Brown; W, Wuhei. a,b,c means a sensor with no common letters significantly differs (*p* < 0.05) (*n* = 9).

Electronic tongue mimics the human tongue and obtains five tastes, including astringency, bitterness, sourness, saltiness, and umami by five sensors [52]. Sensor AE1 evaluates astringency and aftertaste-astringency (aftertaste-A), sensor C00 tests bitterness and aftertaste-bitterness (aftertaste-B), sensor AAE measures umami and its richness, and sensors CT0 and CA0 were for saltiness and sourness, respectively. The tasteless value was −13 for sourness, −6 for saltiness, and 0 for other tastes caused by reference solutions (potassium chloride and tartaric acid). As shown in Figure 4, the sourness taste, whose value was less than the tasteless value of −13 in egg yolks and albumen from three breeds, was not included in the following analyses. For egg yolks in Figure 4A, H yolks had lower values in bitterness and higher values in saltiness than B yolks (*p* < 0.05). The aftertaste-B of egg yolk was not in discussion, with a value less than zero. For egg albumen in Figure 4B, H albumen had lower values in astringency and higher values in saltiness than B albumen (*p* < 0.05). The aftertaste-A value was also less than zero and not in discussion as sourness. Because saltiness values were almost −6, the main different tastes among three breeds of eggs were bitterness for egg yolks and astringency for egg albumen.

**Figure 5:**
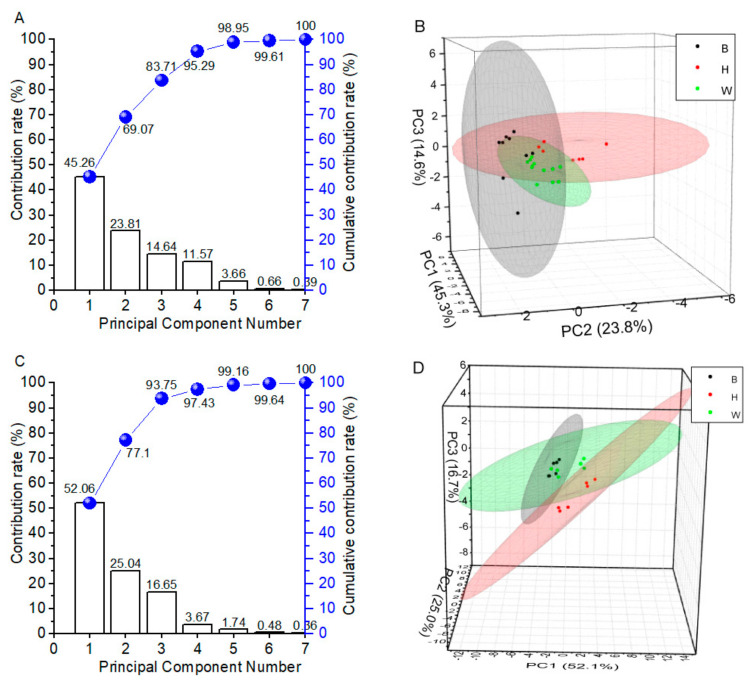
Principal component analysis (PCA) of egg yolks and albumen by electronic tongue (*n* = 9). (**A**) Contribution rate and cumulative contribution rate of yolks. (**B**) PCA score plot of yolks. (**C**) Contribution rate and cumulative contribution rate of albumen. (**D**) PCA score plot of albumen. B, Beinong No.2; H, Hy-Line Brown; W, Wuhei.

Figure 5 shows the result of PCA applied to electronic tongue of egg yolks and albumen. PC1, PC2, and PC3 accounted for 45.3%, 23.8%, and 14.6% of the total variability for egg yolks, respectively. The egg yolks of B, H, and W could not be well grouped by electronic tongue data processed by PCA. For egg albumen, the total variance explained by the PCA was 93.7%, where PC1, PC2, and PC3 explained 52.1%, 25.0%, and 16.7%, respectively. The egg albumen of B, H, and W also could not be grouped by PCA of electronic tongue data. As mentioned above, PCA as an unsupervised algorithm could not adequately group yolks or albumen from the three breeds based on electronic tongue data.

**Figure 6:**
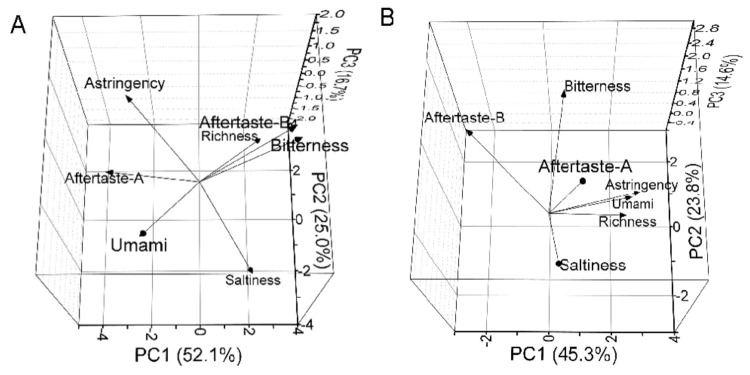
The load coefficient diagrams of electronic tongue by principal component analysis. (**A**) The load coefficient diagrams for yolks. (**B**) The load coefficient diagrams for albumen.

As illustrated in Figure 6 and Table 6, the astringency taste had the highest load coefficient of 0.54 in PC1, which indicates that the astringency had the main positive contribution for egg yolks. The load coefficient of bitterness was the highest value of 0.66 in PC2. The aftertaste-A had the highest load coefficient of 0.54 in PC3. For egg albumen, the load coefficient of bitterness had the highest absolute value, which indicates that aftertaste-A had the main negative contribution in PC1. In PC2 and PC3, the astringency and bitterness had the highest load coefficient. In all, astringency, aftertaste-A, and bitterness dominated the major contributions to taste profiles for egg yolks and bitterness for egg albumen. 

### 3.5. Classification Algorithms 

LDA, fine KNN, linear SVM, and fine tree were adopted to classify yolks, albumen, and whole eggs from three breeds. The classification results of yolks, albumen, and the whole eggs from three breeds were illustrated in Table 7. Based on electronic measurement, the four algorithms all obtained 100% classification accuracy for yolk data. For albumen data measured by electronic nose, LDA, fine KNN, and linear SVM got the best results with 100% classification accuracy, while fine tree got lower classification accuracy with 91.70%. To classify the whole eggs from tree breeds, the yolk data and albumen data based on electronic nose were combined to achieve 100% classification accuracy by four algorithms. 

**Figure 7:**
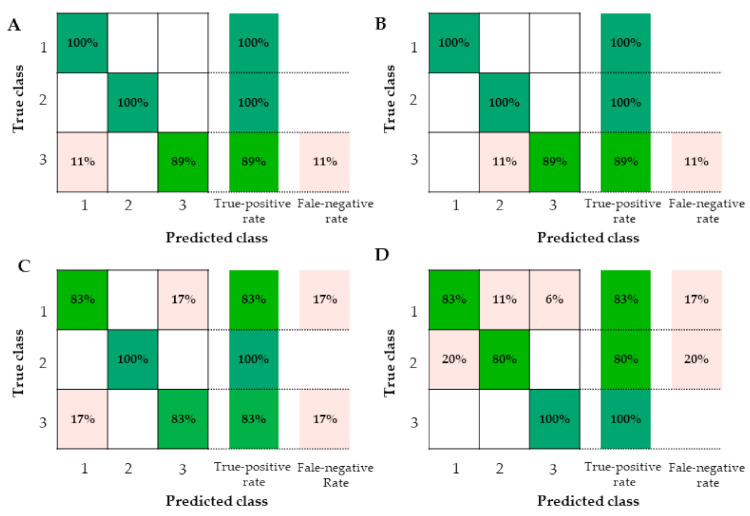
Confusion matrix of electronic tongue for yolks, albumen, and eggs by classification algorithms. (**A**) Confusion matrix for yolks by linear discriminant analysis (LDA). (**B**) Confusion matrix for yolks by fine tree. (**C**) Confusion matrix for albumen by linear discriminant analysis (LDA). (**D**) Confusion matrix for whole eggs by fine K-nearest neighbor (KNN). Number 1, Wuhei (W); Number 2, Beinong No.2 (B); Number 3, Hy-Line Brown (H).

As shown in confusion matrix of Figure 7, it was clear which one misjudged. True-positive rate was the percent of actual positives correctly classified as positives. False-negative rate was the percentage of actual positives incorrectly classified as negatives [51]. For yolk data measured by electronic tongue, LDA and fine tree got better classification accuracy with 96.7% than fine KNN with 90.0% and linear SVM with 73.3%. The LDA (Figure 7A) and fine tree (Figure 7B) got the same true-positive rate and false-negative rate. Number 1, representing W yolks, and number 2, representing B yolks, were correctly classified by LDA and fine tree. A total of 11% of number 3, representing H yolks, were misjudged as number 1, representing W yolks, by LDA and misjudged as number 2, representing B yolks, by fine tree. For albumen data measured by electronic tongue, LDA obtained better classification accuracy with 88.9% than fine KNN with 61.1%, linear SVM with 77.8%, and fine tree with 61.1%. As shown in Figure 7C, only number 2, representing B albumen, were correctly classified based on LDA of electronic tongue data. A total of 17% of W albumen and W albumen were misjudged as each other. For the whole egg classification acquired from yolk data and albumen data of electronic tongue, fine KNN got 87.5% classification accuracy, and the confusion matrix is shown in Figure 7D. Only H eggs were all correctly classified. W eggs had 11% misjudgment rate as B eggs and 6% misjudgment rate as H eggs. A total of 20% of B eggs were misjudged as W eggs. From the above analyses, four algorithms had different classification results for various detection parameters and measurement methods. LDA is a fast, easy-to-interpret discriminant classifier that creates linear boundaries between classes. Fine KNN is a nearest-neighbor classifier that makes finely detailed distinctions between classes, with the number of neighbors set to 1. Linear SVM, the easiest SVM to interpret, is a support vector machine that makes a simple linear separation between classes using the linear kernel. Fine tree is a decision tree with many leaves that makes many fine distinctions between classes and was carried out with 100 maximum number of splits and Gini’s diversity index as Split criterion. Three-fold cross-validation was set to protect against overfitting by partitioning the data set into folds and estimating accuracy on each fold. 

In classification algorithms, the total data of electronic nose and electronic tongue were analyzed without considering sensitive composition or content corresponding to the sensors. This saved calculation and analysis time, ensuring the accuracy of classification results and avoiding impact of specific substance misjudgment. For electronic nose data, LDA, fine KNN, and linear SVM algorithms all obtained 100% accuracy for egg yolks, albumen, and eggs based on electronic sensory qualities. The classification results proved that the three breeds of eggs could successfully discriminated by electronic nose combined with proper algorithms.

## 4. Conclusions

As sensory qualities, the texture, smell, and taste parameters of egg yolks and albumen from B, H, and W were compared and discriminated. TPA results illustrated the hardness, adhesiveness, springiness, and chewiness but not cohesiveness were not significantly different in egg yolks and albumen from three breeds (*p* > 0.05). Electronic nose detection found eight sensors for egg yolks, and all 10 sensors for egg albumen had significant differences, and the sulfur and aromatic content of yolks and albumen from B eggs was obviously higher than that of H and W eggs (*p* < 0.05). Based on electronic nose data, PCA could adequately group egg yolks and albumen from different breeds, and LDA, fine KNN, and linear SVM algorithms got best results, with 100% classification accuracy for yolks, albumen, and the whole eggs. Electronic tongue measurement indicated that bitterness and astringency was the main different taste for yolks and albumen from three breeds. In classification models of electronic tongue, LDA and fine tree obtained 96.7% classification accuracy for yolks, LDA got 88.9% classification accuracy for albumen, and fine KNN achieved 87.5% classification accuracy for the whole eggs. In conclusion, B, H, and W eggs had main differences in smells and could be successfully discriminated by LDA, fine KNN, and linear SVM algorithms based on electronic nose, which is more sensitive to differences in yolks, albumen, and the whole eggs. The results also elucidate methods for identifying characteristics and classifying multiple complex samples in the future. 

## Figures and Tables

**Table 1:** The 10 sensors, sensitive characteristics, and reference volatiles of electronic nose.

Number	Sensors	Sensitive Characteristics	Reference Volatiles
S1	W1C	Aromatic	Toluene, 10 ppm
S2	W5S	Nitrogen oxides	NO_2_, 1 ppm
S3	W3C	Ammonia	Benzene, 10 ppm
S4	W6S	Hydrogen	H_2_, 100 ppb
S5	W5C	Alkane	Propane, 1 ppm
S6	W1S	Methane	CH_3_, 100 ppm
S7	W1W	Sulfur	H_2_S, 1 ppm
S8	W2S	Alcohol, aromatic	CO, 100 ppm
S9	W2W	Aromatic, sulfur organic	H_2_S, 1 ppm
S10	W3S	High concentrations > 100 ppm	CH_3_, 100 ppm

**Table 2:** Egg quality for three breeds of eggs.

Items	B ^1^	H ^2^	W ^3^	SEM ^4^	*p* Value
Color	9.80 ^a^	6.50 ^b^	9.10 ^a^	0.35	0.00
Weight (g)	55.45 ^a^	54.86 ^a^	44.97 ^b^	1.09	0.00
Height (mm)	5.55 ^ab^	6.38 ^a^	4.76 ^b^	0.29	0.03
HU	74.38	78.63	72.22	2.21	0.40

^a,b^ Means within a row with no common superscripts significantly differ (*p* < 0.05) (*n* = 10). ^1^ B, Beinong No.2; ^2^ H, Hy-Line Brown; ^3^ W, Wuhei; ^4^ SEM, standard error of mean.

**Table 3:** Texture properties in egg yolks from three breeds.

Items	B ^1^	H ^2^	W ^3^	SEM ^4^	*p* Value
Hardness (N)	3.02	2.99	3.13	0.14	0.92
Adhesiveness (N.mm)	0.07	0.08	0.07	0.00	0.69
Cohesiveness (Ratio)	0.58 ^a^	0.51 ^ab^	0.47 ^b^	0.02	0.07
Springiness (mm)	4.72	5.14	4.80	0.13	0.43
Chewiness	8.46	8.06	6.95	0.61	0.61

^a,b^ Means within a row with no common superscripts significantly differ (*p* < 0.05) (*n* = 10). ^1^ B, Beinong No.2; ^2^ H, Hy-Line Brown; ^3^ W, Wuhei; ^4^ SEM, standard error of mean.

**Table 4:** Texture properties in egg albumen from three breeds.

Items	B ^1^	H ^2^	W ^3^	SEM ^4^	*p* Value
Hardness (N)	5.20	7.41	7.71	0.48	0.06
Adhesiveness (N.mm)	0.05	0.05	0.04	0.00	0.37
Cohesiveness (Ratio)	0.60 ^b^	0.59 ^b^	0.65 ^a^	0.01	0.04
Springiness (mm)	4.62	5.40	4.28	0.29	0.28
Chewiness	14.44	23.98	22.24	2.17	0.16

^a,b^ Means within a row with no common superscripts significantly differ (*p* < 0.05) (*n* = 10). ^1^ B, Beinong No.2; ^2^ H, Hy-Line Brown; ^3^ W, Wuhei; ^4^ SEM, standard error of mean.

**Table 5:** Electronic nose-load coefficients of egg yolks and albumen by principal component analysis (PCA).

Sensors	Yolk			Albumen		
	PC1 ^1^	PC2 ^2^	PC3 ^3^	PC1	PC2	PC3
W1C	−0.38	0.06	0.25	−0.37	−0.14	−0.06
W5S	0.39	−0.08	0.05	0.36	−0.15	−0.01
W3C	−0.38	0.14	0.1	−0.38	0.03	−0.02
W6S	0.1	0.37	0.68	0.12	0.49	0.78
W5C	−0.38	0.16	0.02	−0.38	0.03	−0.02
W1S	0.26	0.52	−0.28	0.25	0.41	−0.53
W1W	0.36	−0.25	0.2	0.32	−0.33	0.16
W2S	0.22	0.55	−0.36	0.16	0.57	−0.15
W2W	0.36	−0.26	0.14	0.33	−0.32	0.14
W3S	0.18	0.33	0.44	0.35	−0.05	−0.18

^1^ PC1, the first principal component. ^2^ PC2, the second principal component. ^3^ PC3, the third principal component.

**Table 6:** Electronic tongue load coefficients of egg yolks and albumen by principal component analysis (PCA).

Sensors	Yolk			Albumen		
	PC1 ^1^	PC2 ^2^	PC3 ^3^	PC1	PC2	PC3
Astringency	0.54	0.16	0.01	−0.36	0.52	0.16
Aftertaste-A	0.21	−0.19	0.71	−0.48	0.11	−0.06
Bitterness	0.1	0.66	0.39	0.45	0.02	0.47
Aftertaste-B	−0.43	0.36	0.39	0.43	0.11	0.43
Richness	0.46	−0.03	0.03	0.34	0.49	−0.29
Saltiness	0.08	−0.57	0.42	0.29	−0.42	−0.56
Umami	0.5	0.2	−0.13	−0.26	−0.54	0.41

^1^ PC1, the first principal component. ^2^ PC2, the second principal component. ^3^ PC3, the third principal component.

**Table 7:** Classification accuracy (%) of electronic sensory qualities from three breeds of eggs based on different algorithms.

Classification Algorithms	Electronic Nose			Electronic Tongue		
	Yolk	Albumen	Egg	Yolk	Albumen	Egg
LDA ^1^	100%	100%	100%	96.7%	88.9%	58.3%
Fine KNN ^2^	100%	100%	100%	90.0%	61.1%	87.5%
Linear SVM ^3^	100%	100%	100%	73.3%	77.8%	66.7%
Fine tree	100%	91.7%	100%	96.7%	61.1%	64.6%

^1^ LDA, linear discriminant analysis; ^2^ KNN, k-nearest neighbor; ^3^ SVM, support vector machine.

## Data Availability

All data included in this study are available by contacting with the corresponding author.
